# Successful Treatment of Peripheral Nerve Sheath Tumor-related Pain with Perineural Steroid Injection: a case report

**DOI:** 10.1016/j.inpm.2024.100394

**Published:** 2024-02-28

**Authors:** David S. Jevotovsky, Elizabeth Callahan, Jina Libby, Salvador E. Portugal

**Affiliations:** NYU Grossman School of Medicine, Rusk Rehabilitation, 333 East 28th Street, New York, NY 10016, USA

**Keywords:** Pain, Injections, Neurofibromatosis 1, Ultrasonography

## Abstract

Pain associated with Neurofibromatosis Type 1 (NF1) is poorly understood. To date, no treatment options have been approved for NF1-related pain. We present the case of a young female NF1 patient with intermittent buttock pain radiating down the leg who presented with positive dural tension signs. The patient was diagnosed with neurofibroma sciatic nerve compression, which was successfully managed with ultrasound-guided perineural steroid injection. There is sparse literature regarding the efficacy of ultrasound-guided perineural steroid injection in NF1 patients for treatment of benign peripheral nerve sheath tumor compressions. This case describes the utility of perineural steroid injections for symptomatic relief of NF1 neurofibroma-related pain. Perineural steroid injections should be considered when neurofibroma-related pain fails to respond to other conservative treatment. Steroid injections provide an alternative to oral medicinal management and avoid the often morbid risks of surgical intervention.

## 1 Introduction

NF1 is an autosomal dominant, neuroectodermal condition that affects muscle, bone and nerve composition due to increased Ras proto-oncogene signaling. Pain in NF1 patients is currently poorly understood. Estimates of NF1 pain prevalence range from 29% to 70%, depending on the report [1]. As many as 25–50% of patients with NF1 develop peripheral neurofibromas [2], leading to heterogeneous presentations of pain in NF1 patients [3]. Non-headache NF1 pain is commonly radicular in nature secondary to central or peripheral nerve involvement [4].

**Fig. 1:**
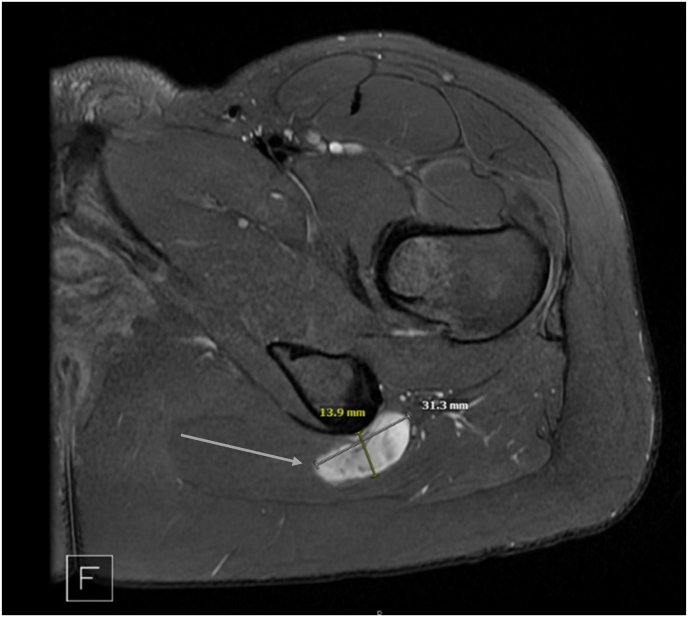
Lumbar Spine MRI. Lumbar Spine MRI revealing 3.1 cm nerve sheath tumor in the ischiogluteal space at the level of the inferior gemellus with mass effect on the sciatic and inferior gluteal nerves as indicated by the arrow.

**Fig. 2:**
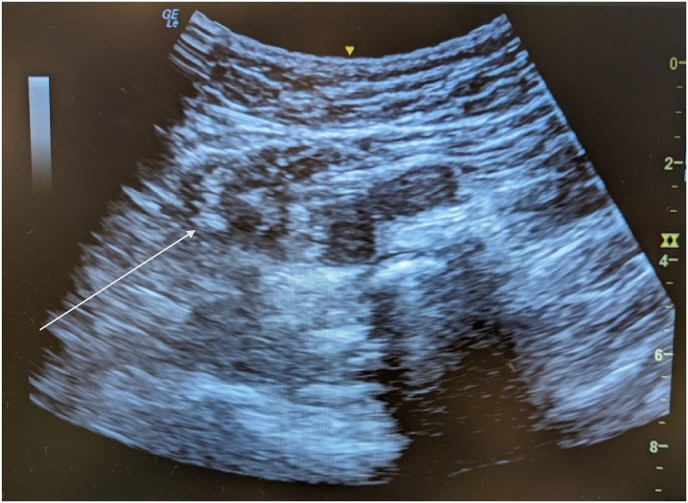
Ultrasound-guided injection. Left sciatic neurofibroma (indicated by arrow sign) visualized on ultrasound during perineural steroid injection.

There are presently no approved treatments for NF1-related pain. Patients have historically turned to management with either non-steroidal anti-inflammatory drugs, acetaminophen, alone or in combination with prescription pain medicines such as opioids, anticonvulsants, antidepressants or topical lidocaine patches [3].

We present a case of perineural steroid injection for the treatment of NF1 pain, which, to our knowledge, has not been described.

## 2 Case presentation

A 21-year-old female with no known past medical history presented to the outpatient setting complaining of several years of left-sided buttock pain radiating into the left leg. She described the pain as a dull, aching throb that was intermittent and nonprogressive in nature. The pain worsened at night, which the patient attributed to periods of prolonged sitting or lying down. Positional changes temporarily alleviated her symptoms. She denied known trauma or injury to the area, fever, chills, bowel dysfunction, bladder dysfunction, saddle anesthesia, and unexplained weight loss.

Physical examination was notable for tenderness over the left piriformis region, 3+ bilateral lower extremity deep tendon reflexes, positive straight leg raises and slump tests, and two beats of ankle clonus bilaterally. Babinski's sign, FABER and FADIR test were all negative. The patient had full strength and sensation was intact throughout.

Lumbar radiculopathy was highest on the differential given the patient's radicular complaints and positive dural tension signs. Piriformis syndrome was also considered given the tenderness to palpation and reproducibility of pain with muscular stretch. Lumbar and left hip MRI were ordered for further evaluation. The patient was also prescribed a trial of physical therapy.

Lumbar MRI showed no evidence of canal or foraminal stenosis but revealed multiple rounded T2 hyperintense lesions with targetoid appearance along the extraforaminal nerve roots. Similarly appearing lesions were noted in the bony pelvis on the hip MRI, including a 3.1 cm lesion in the ischiogluteal space with mass effect on the inferior gluteal nerve (see Fig. 1). A 7.1 cm plexiform lesion was noted along the right iliac crest and other smaller lesions were found within the left sciatic, femoral and obturator nerves. Considering the lack of evidence of lumbosacral nerve root impingement on diagnostic imaging, and the reproduction of symptoms with sonographic palpation of the large 3.1 cm lesion, it was concluded that this lesion was the source of her symptoms.

At two-month follow-up, the pain had only mildly improved with physical therapy to a level of 6 out of 10 on the Numerical Pain Rating Scale (NPRS). She continued to deny focal weakness or bowel/bladder dysfunction, and the physical exam was unchanged. Upon further investigation, the patient divulged a medical history of neurofibromatosis (NF1) diagnosed 3 years prior. Patient agreed to ultrasound-guided left ischiogluteal perineural steroid injection for symptomatic relief. The left sciatic neurofibroma was visualized on ultrasound with a curvilinear probe (see Fig. 2). A 25-gauge 3.5-inch needle was attached to a 6cc syringe containing 2cc of 0.5% Lidocaine and 1 cc of Dexamethasone Sodium Phosphate (10 mg/ml) and directed toward the ischiogluteal lesion. The needle was visualized in-plane and directed to the target from a lateral to medial approach. After needle tip placement was confirmed on ultrasound, the perineural injection was administered. Injection was performed at the level of the inferior gemellus with the transducer positioned 90° transverse to the body's midline. Dexamethasone was chosen given its non-particulate characteristics. A small volume of injected material was utilized to balance the medications' therapeutic effects while avoiding further mass effect on the nerve. The patient tolerated the procedure well. Post-procedure physical exam demonstrated full motor strength and intact sensation in the bilateral lower extremities. The patient denied any sensory or motor deficits in the sciatic nerve distribution. Significant symptomatic improvement was noted within the first week post-injection with only two mild episodes of pain, and pain had resolved to an NPRS of 0 by one month follow-up. For four months after the perineural steroid injection, the NPRS remained 0 out of 10.

## 3 Discussion

This patient exemplifies how non-headache NF1 pain is commonly radicular in nature secondary to central or peripheral nerve involvement [4].

There are presently no approved treatments for NF1-related pain. Though patients have historically turned to management with either non-steroidal anti-inflammatory drugs, acetaminophen, alone or in combination with prescription pain medicines [3]. New medications are under investigation. Much of the pharmacologic treatment options for NF1 pain remain in clinical and preclinical stages, including those that target mTOR or MEK pathways for malignant peripheral nerve sheath tumors [3].

Large symptomatic lesions like this patient's neurofibroma are at times managed with surgical excision, but surgical intervention comes with risks [5]. Unlike solitary neurofibromas, plexiform neurofibromas risk significant morbidity from surgical management [6]. Given that surgical goals for these tumors are symptomatic management, surgical resection of plexiform lesions is restricted to tumors that cause significant impairments.

A few reports document the use of ultrasound-guided peripheral nerve blocks as regional anesthesia prior to surgical removal of lesions in NF1 patients [7,8], but there is sparse literature regarding the efficacy of ultrasound-guided perineural steroid injection. There is some evidence regarding the efficacy of perineural peripheral nerve injections in the setting of carpal tunnel syndrome, ulnar neuropathy, Morton's neuroma; however, to our knowledge this has not been expanded to the treatment of benign peripheral nerve sheath tumors such as neurofibromas found in NF1 [9]. Given this patient's radicular symptoms, peri-sciatic imaging findings, and similarity to known indications, the healthcare team recommended perineural steroid injection which resulted in the successful treatment of the patient's left lower extremity pain.

To our knowledge, this is the first report of pain related to a neurofibroma successfully treated with a perineural steroid injection. It is important for physicians to be aware of NF1 as a possible etiology of non-specific pain that does not respond to conservative measures. In these cases, it is imperative to obtain timely imaging to help make the diagnosis. Steroid injections provide an alternative to chronic medicinal management and avoid the often morbid risks of surgical intervention. More research needs to be conducted to evaluate the efficacy of ultrasound-guided perineural steroid injections for the treatment of benign nerve sheath tumors like those found in NF1 patients.

## Funding

This research did not receive any specific grant from funding agencies in the public, commercial, or not-for-profit sectors.

## Author disclosures

None.

## Availability of data

The data that support the findings of this study are available from the corresponding author upon reasonable request.

## Declaration of competing interest

The authors declare that they have no known competing financial interests or personal relationships that could have appeared to influence the work reported in this paper.

## References

[1] Bellampalli Shreya S., Khanna Rajesh (2019). Towards a neurobiological understanding of pain in neurofibromatosis type 1: mechanisms and implications for treatment. Pain.

[2] Waheed W., Diego F., Lemos D.F., Nathaniel Nelms N., Tandan R. (2016). Multifactorial pathological hip subluxation in neurofibromatosis type-1 (NF1) due to intra-articular plexiform neurofibroma, lumbar radiculopathy and neurofibromatous polyneuropathy. BMJ Case Rep.

[3] Bellampalli Shreya S., Khanna Rajesh (2019). Towards a neurobiological understanding of pain in neurofibromatosis type 1: mechanisms and implications for treatment. Pain.

[4] Créange A., Zeller J., Rostaing-Rigattieri S., Brugières P., Degos J.D., Revuz J., Wolkenstein P. (1999). Neurological complications of neurofibromatosis type 1 in adulthood. Brain.

[5] Sieb J.P.M.D., Schultheiss R.M.D. (1992). Segmental neurofibromatosis of the sciatic nerve: case report. Neurosurgery.

[6] Pollack I.F., Colak A., Fitz C., Wiener E., Moreland M., Mulvihill J.J. (1998). Surgical management of spinal cord compression from plexiform neurofibromas in patients with neurofibromatosis 1. Neurosurgery.

[7] Şalvız, Aysu Emine (2018). Use of ultrasound-guided supraclavicular brachial plexus block as an anesthesia technique in a patient with neurofibromatosis type 1: a case report. Agri.

[8] Rocco M.L., Rosenblatt M.A. (2011). Ultrasound-guided peripheral nerve block in a patient with neurofibromatosis. Reg Anesth Pain Med.

[9] Tagliafico Alberto (2010). Peripheral nerves: ultrasound-guided interventional procedures. Semin Muscoskel Radiol.

