# Supplementary material for: Successful Treatment of Peripheral Nerve Sheath Tumor-related Pain with Perineural Steroid Injection: a case report
Source: Interv Pain Med. 2024 Feb 28;3(1):100394. doi: 10.1016/j.inpm.2024.100394 (PMC11372941; doi:10.1016/j.inpm.2024.100394)
Supplement: Multimedia component 1 [file mmc1.pdf]

CARE case report guidelines

CARE Checklist of information to include when writing a case report

(cc) BY-NC-ND

Topic	Item	Checklist item description	Reported on Line
Title	1	The diagnosis or intervention of primary focus followed by the words "case report"	1
Key Words	2	2 to 5 key words that identify diagnoses or interventions in this case report, including "case report"	41
Abstract (no references)	3a	Introduction: What is unique about this case and what does it add to the scientific literature?	30-31
	3b	Main symptoms and/or important clinical findings	31-32
	3c	The main diagnoses, therapeutic interventions, and outcomes	33-36
	3d	Conclusion—What is the main "take-away" lesson(s) from this case?	36-39
Introduction	4	One or two paragraphs summarizing why this case is unique (**may include references**)	51-64
Patient Information	5a	De-identified patient specific information.	68
	5b	Primary concerns and symptoms of the patient.	68-73
	5c	Medical, family, and psycho-social history including relevant genetic information	68,93
	5d	Relevant past interventions with outcomes	91
Clinical Findings	6	Describe significant physical examination (PE) and important clinical findings.	75-78
Timeline	7	Historical and current information from this episode of care organized as a timeline	69-91
Diagnostic Assessment	8a	Diagnostic testing (such as PE, laboratory testing, imaging, surveys).	85-89
	8b	Diagnostic challenges (such as access to testing, financial, or cultural)	N/A
	8c	Diagnosis (including other diagnoses considered)	80-83, 91-95
	8d	Prognosis (such as staging in oncology) where applicable	N/A
Therapeutic Intervention	9a	Types of therapeutic intervention (such as pharmacologic, surgical, preventive, self-care)	94-99
	9b	Administration of therapeutic intervention (such as dosage, strength, duration)	96
	9c	Changes in therapeutic intervention (with rationale)	91-99
Follow-up and Outcomes	10a	Clinician and patient-assessed outcomes (if available)	97-99
	10b	Important follow-up diagnostic and other test results	91-99
	10c	Intervention adherence and tolerability (How was this assessed?)	97
	10d	Adverse and unanticipated events	97-99
Discussion	11a	A scientific discussion of the strengths AND limitations associated with this case report	106-108
	11b	Discussion of the relevant medical literature **with references**.	118-126
	11c	The scientific rationale for any conclusions (including assessment of possible causes)	118-126
	11d	The primary "take-away" lessons of this case report (without references) in a one paragraph conclusion	128-134
Patient Perspective	12	The patient should share their perspective in one to two paragraphs on the treatment(s) they received	N/A
Informed Consent	13	Did the patient give informed consent? Please provide if requested	Yes ☒ No ☐
